# The Book World of Medicine and Science

**Published:** 1896-07-04

**Authors:** 


### The Book World of Medicine and Science.

Year-book of the Scientific and Learned Societies of Great Britain and Ireland. (London: Charles Griffin and Co. 1896. Price 7s. 6d.)

It is only by turning over the pages of such a volume as this that one can gain any idea of the widespread love of science which permeates the land we live in. Perhaps it would be too much to say that all the societies entered on this long list are scientific in their methods, or show deep learning in their proceedings; but they exist, and it is a matter of no small interest to find, as is shown by this hand-book, that in almost every town men are found sufficiently interested in such matters to band themselves together for their investigation and discussion. To all such it must be a matter of much importance to know what is being done by their compeers in similar fields, and we can quite understand how useful such a hand-book as this must be to them. It is not a mere list, but contains a concise review of the history, organisation, and conditions of membership of the various societies, together with a chronicle of the work which they have done during the past year, and of the various official changes which have taken place. Altogether, it is a most convenient work of reference.

Biological Experimentation. By Sir B. W. Richardson, M.D., F.R.S. (London: George Hill and Sons.)

Sir Benjamin Ward Richardson represents in his own person two personalities: the personality of the man of science, and the personality of the average man of culture. On the question of Biological Experimentation he is peculiarly fitted to speak. He has been for many years an experimenter; he has also been a humanitarian. He knows what is to be gained by experimentation; he knows also the cost of the acquisition. The subject is admittedly difficult; at any rate to the man of normal human feeling. No doubt there are persons, especially among young men who know nothing of the world, and who are not capable of thinking anything beyond the range of their own specialty, who will look upon Sir Benjamin Richardson as a weak-kneed physiologist, who may be treated with indifference by physiologists of less sensitive minds. But such persons have yet much to learn. When they have battled as long with the ever-present problems of science and humanitarianism as Sir Benjamin Richardson has, they will speak with much more modesty. Any reader of Sir Benjamin Richardson's book may discover almost immediately that the author has an innate dislike of experiments on living animals which produce terror and inflict pain. This is surely an honourable condition of mind. At the same time Sir Benjamin does not agree with those who hold that painful experiments on living animals should never be made. With true philosophic insight he insists that such experiments are being made all the day long and every day in the great laboratory of Nature, and that without the slightest compunction or regard for consequences. What Nature does men may do, it is urged. But, on the other hand, Sir Benjamin thinks it a higher *rôle* to be a repairer of Nature's ravages than to be an imitator; and therein we agree with him. What, then, are the general conclusions reached by this scientific master on this great and urgent question? The general conclusions are that man may experiment on living animals if he can do so profitably to science, to humanity, and to animals themselves. But this right of man is not an unconditional right. It is a right, in the first place, for the competent, and for the competent only. Crude "scientasters," to coin a word, should not be permitted to make painful and terrorising experiments on living animals. Masters only, and men of high science and proved humanity, should be endowed with this fearful privilege. There is, however, as Sir Benjamin points out, a field in which any man of inquiring mind may become an original investigator. That field is the vast laboratory of Nature, in which, among plants and shrubs, among fish and fowl, among four-footed creatures and human beings, innumerable experiments are unceasingly being carried out. A few only may go to the physiological laboratory, because to a few only is given the competent philosophy and the large humanity demanded of the experimenters on living animals. But to the open laboratory of Nature all may go; and Sir Benjamin Richardson holds that Nature, if patiently, persistently, and intelligently interrogated, will teach us more and of more value than the physiological laboratory. The little work is of surpassing interest at the present moment, and the price —2s. 6d.—places it within reach of all.

The Diagnosis and Treatment of Diseases of the Rectum. By William Allingham, F.R.C.S., and Herbert W. Allingham, F.R.C.S. (London: Baillière, Tindall, and Cox. Sixth edition. 1896. Price, 12s. 6d.)

We are by no means admirers of that tendency to specialism which would allocate to different practitioners the treatment of the ailments of the different portions of the body. It is clear enough, however, that it is a tendency which can only be met by the general practitioner appropriating to his own use the knowledge of the specialist. A very slight acquaintance with medical literature is sufficient to show how sketchy and superficial are the accounts of many of the more special diseases which are to be found in the general text-books. Such books are, of course, a necessity, as linking together all diseases in one general survey; but if a practitioner wishes for anything like full knowledge on any subject he must appeal to special works, and for this purpose we can strongly recommend the work before us as an admirable compendium of special knowledge in regard to that far too much neglected subject, diseases of the rectum. This book shows in marked degree the advantages which may be gained from conjoint authorship, for while the senior author is a specialist of specialists, the junior, although largely engaged in special work, is a well-known surgeon connected with two large general hospitals in London, and we may add that the influence of the more modern modes of thought are very manifest in its pages. The chapters on cancer of the rectum and on colotomy are admirable and full of hints and directions for rendering such operative proceedings as may be undertaken really helpful to the patient. Under the head of "Fistula" emphasis is laid on the importance, in operating, of dealing thoroughly with all the branching sinuses which may exist; and it is shown how easy it is in passing the director to make a false passage and then to omit the division of a considerable length of the sinus. In regard to the question of operating in the presence of phthisis, attention is drawn to the curious effect which fistula exercises on the mind. "Phthisical patients are usually very hopeful, and are constantly making plans for the future with a happy absence of despondency. But a strong, healthy man who suffers from fistula speedily becomes exceedingly despondent. *A fortiori*, then, if a phthisical patient is told that he has also a fistula which must not be cured, or even treated, although he may scarcely worry about his phthisis, he may be thrown into a state of great perturbation and mental anxiety by the news that he has a fistula which cannot or ought not to be cured." The work throughout is well printed, is written in a clear readable style, and bears evidence on every page of wide practical knowledge of the conditions of which it treats.

